# An m6A-Driven Prognostic Marker Panel for Renal Cell Carcinoma Based on the First Transcriptome-Wide m6A-seq

**DOI:** 10.3390/diagnostics13050823

**Published:** 2023-02-21

**Authors:** Frank Waldbillig, Felix Bormann, Manuel Neuberger, Jörg Ellinger, Philipp Erben, Maximilian C. Kriegmair, Maurice Stephan Michel, Philipp Nuhn, Malin Nientiedt

**Affiliations:** 1 Department of Urology & Urosurgery, University Medical Centre Mannheim, University of Heidelberg, 68167 Mannheim, Germany; 2 Bioinformatics.Expert UG, 12305 Berlin, Germany; 3 Department of Urology & Pediatric Urology, University Medical Centre Bonn, University of Bonn, 53127 Bonn, Germany

**Keywords:** epitranscriptomics, *N*^6^-methyladenosine (m^6^A), METTL3, m6A-reader, MeRip-seq, uromodulin, kidney cancer

## Abstract

To date, only a single transcriptome-wide m6A sequencing study of clear cell renal cell carcinoma (ccRCC) has been reported, with no validation so far. Herein, by TCGA analysis of the KIRC cohort (*n* = 530 ccRCC; *n* = 72 normal), an external expression validation of 35 preidentified m6A targets was performed. Further in-depth expression stratification enabled assessment of m6A-driven key targets. Overall survival (OS) analysis and gene set enrichment analyses (GSEA) were conducted to assess their clinical and functional impact on ccRCC. In the hyper-up cluster significant upregulation was confirmed for NDUFA4L2, NXPH4, SAA1, and PLOD2 (40%) and in the hypo-up cluster for FCHSD1 (10%). Significant downregulation was observed for UMOD, ANK3, and CNTFR (27.3%) in the hypo-down cluster and for CHDH (25%) in the hyper-down cluster. In-depth expression stratification showed consistent dysregulation in ccRCC only for 11.67%: NDUFA4L2, NXPH4, and UMOD (NNU-panel). Patients with strong NNU panel dysregulation had significantly poorer OS (*p* = 0.0075). GSEA identified 13 associated and significantly upregulated gene sets (all *p*-values < 0.5; FDR < 0.25). External validation of the only available m6A sequencing in ccRCC consistently reduced dysregulated m6A-driven targets on the NNU panel with highly significant effects on OS. Epitranscriptomics are a promising target for developing novel therapies and for identifying prognostic markers for daily clinical practice.

## 1. Introduction

Renal cell carcinoma (RCC) has increasing incident rates and accounts for approximately 5% of all oncological diagnoses [1]. It is the 12th most prevalent cancer and caused about 180,000 deaths worldwide in 2020 (https://gco.iarc.fr/, accessed on 1 October 2022). RCC tumour stage and grading has a profound effect on mortality. Despite new systemic therapies, while patients in UICC stage 1 have a favourable 5-year survival rate of 98.5%, the rate decreases to 18.3% for patients in stage 4 [2]. Although numerous molecular targets have been studied, no molecular prognostic biomarkers are clinically used to predict RCC overall survival [3]. While epigenetic methylation has been widely discussed as a possible tracing tool, epitranscriptomic markers have received little attention [4].

First described in 1974, m6A methylation is the most abundant internal mRNA modification in eukaryotes [5]. However, little was known about its cellular role until 2012, when the first transcriptome-wide detection methodology showed that methylated adenosine bases can influence the cell cycle and are enriched in long coding exons sections, in proximity of stop codons, or in the 3’UTR region [6,7]. According to the DRACH consensus, m6A sites usually occur in a sequence-specific manner: D (A, G, or U), R (A or G), A^m^, C, H (A, C, or U) [8,9]. m6A methylation is a dynamic process, thus m6A marks are reversibly adjusted to physiological and pathological cell conditions. The m6A enzyme complex methylates or demethylates adenosine in mRNA during transcription. The complex is formed by m6A writers (methylation unit), m6A erasers (demethylation unit), and m6A readers (interaction proteins) [10]. METTL3 as main m6A-writer is essential for cell cycle and cell survival [11].The m6A readers regulate gene expression via the methylated target transcripts. m6A modifications can facilitate (pre-)mRNA splicing, influence mRNA stability, direct nuclear export, affect translation regulation, and can regulate interaction with non-coding RNA [12,13,14,15,16,17]. The involvement in cellular signalling pathways suggests that aberrant m6A modifications may be implicated in disease aetiology. Indeed, a dysregulated m6A complex has been associated with various types of cancer, including RCC [18,19]. However, the role of the m6A modification in RCC is complex and not yet well defined. While many studies have focused on the m6A complex itself, there has been only limited research on m6A-targeted transcripts in RCC [20,21,22]. Chen et al. have reported the only transcriptome-wide m6A sequencing study on clear cell RCC (ccRCC) [23]. Their analysis defined 35 genes with significant m6A differences and associated changes in mRNA abundance compared to normal renal tissue samples. Based on methylation levels and mRNA expression dysregulation of the m6A target genes, a classification of hyper-up, hypo-down, hyper-down, and hypo-up transcripts was established. There has been no external validation of these findings.

The aim of this study was to verify the 35 previously identified m6A target transcripts and investigate their associated distribution patterns in an established, publicly available ccRCC cohort. Using gene set enrichment analysis (GSEA), we further investigated their potential regulatory functions in ccRCC. Finally, we describe a possible m6A-related prognostic marker panel for ccRCC.

## 2. Materials and Methods

*TCGA expression analysis.* Expression data analysis was performed on the publicly available ccRCC TCGA-KIRC cohort. Expression and clinical data were accessed and downloaded via the Genomic Data Commons data portal (https://portal.gdc.cancer.gov, accessed on 1 March 2022). Expression data were used in the form of HT-seq raw counts. Data were checked for accuracy. Replicates of multiply occurring samples were excluded. The data were downloaded in March of 2022. Data from 530 ccRCC samples and 72 normal renal tissue samples were included in the final expression analysis. The analysis was carried out using R software (v. 4.1.3). DESeq2 (v. 1.34.0) was used for normalisation of raw sequencing data. Expression differences between ccRCC and normal renal tissue were calculated by the Mann–Whitney-U test and quantified by fold change calculations. Heat maps for the four distribution patterns were generated with the pheatmap R package (v. 1.0.12) by normalising raw counts of the 35 genes identified by Chen et al. using DESeq2, adding a pseudocount, and log2 transformation. Genes from normal and tumour samples were hierarchically clustered and sorted according to their normalised mean gene expression. An adjusted *p*-value < 0.05 was assumed for significance. Fold change was considered significant if <0.5 or >2. Boxplots were generated for detailed visualisation of the three key m6A-driven key target genes NDUFA4L2, NXPH4, and UMOD.

To compare expression levels of the major m6A readers to the overall expression level in ccRCC samples, log2-transformed normalised counts after adding a pseudocount were used. Therefore, the mean gene expression for the major m6A readers was first calculated for each sample. For comparison, the overall mean gene expression was calculated for each sample by averaging all mean gene expressions. The results were visualised as boxplots.

*In-depth Expression Stratification.* In the available TCGA-KIRC cohort (*n* = 602), matching ccRCC and normal sample pairs were identified (*n* = 72) and the simple fold change was calculated for each target gene and each pair. The expression differences between ccRCC and normal renal tissue samples were used to binarise the results. Samples with a fold change for the respective genes >1.5 were scored as 1 and samples with a fold change <0.66 were scored as −1. Genes with irrelevant expression differences between the respective samples (fold change range between 0.66–1.5) were neutralised. Based on the binarised results, the respective samples were homogenised into two groups. Group 1 comprised all samples in which none of the seven significantly validated m6A-driven hyper-up and hypo-down m6A genes showed a difference in expression pattern compared to the Chen et al. results. Group 2 comprised all samples in which at least one of the seven genes deviated in the direction of expression from the expected regulation. A homogeneous and consistent expression pattern was defined as <10% expression variation in the corresponding sample cohort.

*Overall survival analysis.* The overall TCGA-KIRC cohort was analysed for absolute mRNA expression of NDUFA4L2, NXPH4, and UMOD. For each key target, all samples were grouped equally into high and low expressing groups. Each sample which was classified either in the NDUFA4L2/NXPH4-high expressing group or the UMOD-low expressing group was stratified into a characteristic m6A-driven cluster we have designated as the NNU panel (*n* = 76). For comparison, all patient samples that did not meet the stratification criteria were also grouped together (*n* = 454). Overall survival was defined as the endpoint. Survival times were compared using the log-rank test and Cox regression analysis. Survival curves were graphed as Kaplan–Meier plot.

*Gene set enrichment analysis.* Analysis was performed using MSigDB GSEA (v. 4.0.1) (http://www.gsea-msigdb.org, accessed on 1 June 2022) with normalised counts. Patient samples were divided into two groups.

One group contained samples belonging to the top 50 percentile of upregulated NDUFA4L2 as well as NXPH4 expression and at the same time to the lowest 50 percentile of downregulated UMOD expression (*n* = 76). The other group was the inverse correlate of this with samples of the bottom 50 percentile of NDUFA4L2 and NXPH4 expression and top 50 percentile of UMOD expression (*n* = 72). The hallmark gene set “h.all.v7.0.symbols.gmt” was used with inclusion of 34,125 genes. Gene sets with a normalised *p*-value < 0.05 and a false discovery rate (FDR) < 0.25 were considered significant.

## 3. Results

### 3.1. Verification of Dysregulated m6A-Driven Target Genes in ccRCC

**Figure 1:**
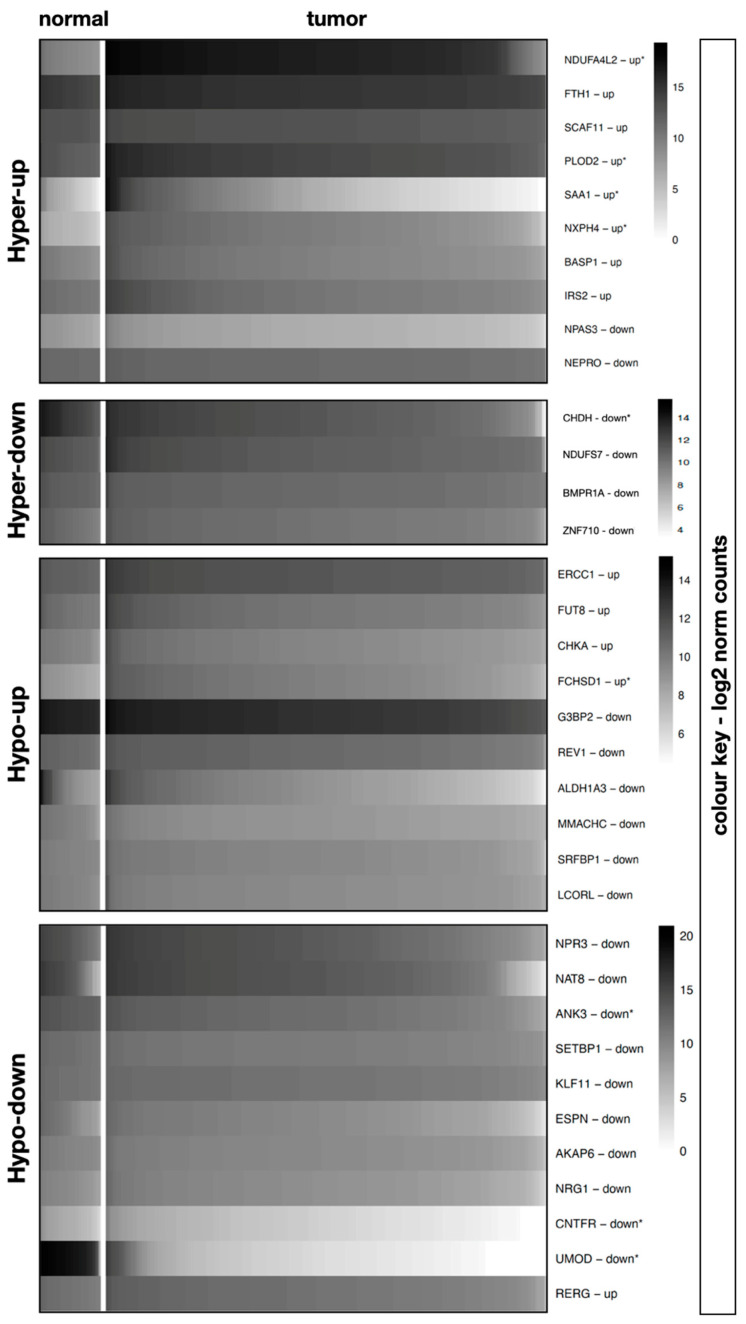
Heatmap based on TCGA-KIRC cohort expression data. The “hyper-up”, “hyper-down”, “hypo-up”, and “hypo-down” groups were defined by Chen et al. (21) and based on potential m6A-driven expression patterns. Genes were hierarchically clustered and samples were subsequently arranged based on gene expression. * Indicates that the TCGA mRNA dysregulation pattern matched the pattern described by Chen and meets significance criteria (*p*-value < 0.05 and FC > 2 or <0.5).

Initially, the four dysregulated m6A-driven expression patterns described by Chen et al. were reviewed via TCGA-KIRC cohort expression analysis. Of the 10 genes identified as hyper-up, 8 were confirmed to have high expression in the TCGA-KIRC cohort. Of these, only four (40%) genes, namely NDUFA4L2 (fc 55.55; adjusted *p*-value < 0.0001), NXPH4 (fc 23.17; adjusted *p*-value < 0.0001), SAA1 (fc 21.51; adjusted *p*-value < 0.0001), and PLOD2 (fc 3.45; adjusted *p*-value < 0.0001), met the significance criteria. Low expression for all four genes designated as hyper-down were confirmed in the TCGA-KIRC, but only CHDH was significant (fc 0.40; adjusted *p*-value < 0.0001). Of the genes classified in the hypo-down panel, downregulation in TCGA-KIRC was verified in 10 of the 11 genes and 3 (27.3%) were significant: UMOD (fc 0.0028; adjusted *p*-value < 0.001), ANK3 (fc 0.39; adjusted *p*-value < 0.0001), and CNTFR (fc 0.40; adjusted *p*-value < 0.0001). The reported dysregulation of KLF11 (fc 0.86; adjusted *p*-value = 0.0053) and NPR3 (fc 0.96; adjusted *p*-value = 0.8058) could not be validated. In the hypo-up gene panel, high expression was confirmed for 4 of the 10 transcripts, of which only FCHSD1 (10%) (fc 2.46; adjusted *p*-value < 0.0001) showed significant overexpression. Thus, significant and consistent dysregulation in ccRCC was confirmed for 25.71% of the m6A targets identified by meRIP-seq. Figure 1 illustrates the TCGA-ccRCC expression differences between normal and ccRCC samples for the 35 predefined genes. Detailed TCGA-KIRC expression data of all genes can be found in Table 1.

### 3.2. Significantly Lower Overall Survival Was Found for NNU Panel Subjects

**Figure 2:**
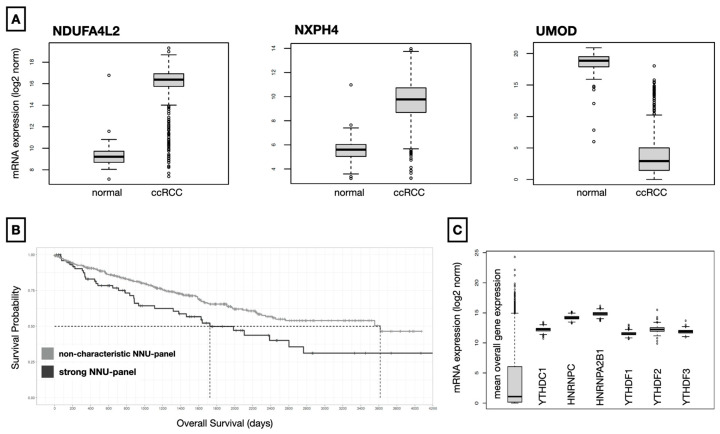
Expression analysis of the TCGA-KIRC cohort. (**A**) Boxplots of the NNU panel expression differences between clear cell renal cell carcinoma (ccRCC) and normal renal tissue. NDUFA4L2 (fc 55.55; adjusted *p*-value < 0.0001) and NXPH4 (fc 23.17; adjusted *p*-value < 0.0001) showed significant upregulation. A significant downregulation was observed for UMOD (fc 0.0028; adjusted *p*-value < 0.001). (**B**) Kaplan–Meier plot visualising the impact of a characteristic m6A-driven NNU panel on overall survival. Affected patients had a significantly poorer outcome (*p* = 0.0075, log-rank). (**C**) Expression profiles of the major m6A readers in ccRCC. YTHDC1, HNRNPC, HNRNPA2B1, and YTHDF1-3 were all strongly expressed in ccRCC compared to the mean overall gene expression of the cancerous tissue.

Since Chen et al. have shown that mRNA expression levels of m6A-driven target transcripts in ccRCC tend to show a positive correlation with their respective m6A methylation level, we performed a more in-depth stratification of clinically relevant m6A-driven target genes in the TCGA-KIRC cohort based on the dysregulated hyper-up and hypo-down distribution patterns. By binarising the expression differences of the matching and significantly validated dysregulated hyper-up and hypo-down genes in 72 TCGA-ccRCC samples with corresponding normal controls, a consistent and homogeneous expression pattern (expression mismatch < 10%) was confirmed for three (11.67%) of the 35 predefined m6A genes, namely NDUFA4L2, NXPH4, and UMOD (Supplementary Figure S1). Expression boxplots of the three validated m6A-driven key genes in TCGA-ccRCC are shown in Figure 2A. All cancer patients in the overall TCGA-KIRC cohort that simultaneously showed significantly high expression of NDUFA4L2 and NXPH4 (hyper-up) and significantly low UMOD expression (hypo-down) were identified and grouped together in the NNU panel. This panel was then compared to the remaining TCGA ccRCC patients (non-characteristic NNU panel). In subsequent survival time analyses, patients in the NNU panel showed a highly significant (*p* = 0.0075, log-rank) worse overall survival. In case of a characteristic NNU panel, 50% survival probability is already reached after 1724 days, whereas it only occurs after 3615 days for an uncharacteristic NNU panel (hazard ratio 0.61, *p*-value 0.008, 95% CI 0.42–0.88). Figure 2B visualises the survival differences in a Kaplan–Meier plot.

Expression levels of the major m6A readers in ccRCC were also examined. YTHDC1, HNRNPC, HNRNPA2B1, YTHDF1, YTHDF2, and YTHDF3 all showed significantly high expression in ccRCC (all *p*-values < 0.0001) compared to the average overall gene expression in the tumour tissue (Figure 2C).

### 3.3. Enrichment of Essential Signalling Pathways in ccRCC Is Co-Determined by NDUFA4L2, NXPH4, and UMOD

**Figure 3:**
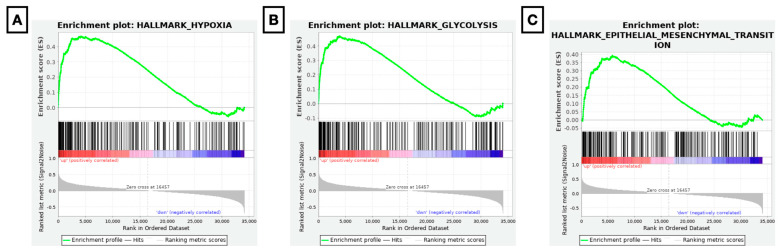
Gene set enrichment analysis plots of a characteristic m6A-driven NNU panel. Dysregulation of the NNU panel was found to be associated with critical biological processes, including (**A**) hypoxia (NES 2.26; *p*-value < 0.0001; FDR < 0.0001), (**B**) glycolysis (NES 2.25; *p*-value < 0.0001; FDR < 0.0001), and (**C**) epithelial-to-mesenchymal transition (NES 1.89; *p*-value < 0.0001; FDR < 0.001).

GSEA was performed to better understand the functional relevance of the NNU panel on ccRCC carcinogenesis. Patients with the strongest NNU panel dysregulation were compared to non-characteristic NNU panel patients. In total, 50 gene sets with a total of 34,125 genes were included in the analysis. Significant upregulation (*p*-value < 0.5; FDR < 0.25) of 13 hallmark gene sets was observed (Table 2). Cellular processes such as hypoxia (NES 2.26; *p*-value < 0.0001; FDR < 0.0001), glycolysis (NES 2.25; *p*-value < 0.0001; FDR < 0.0001), and epithelial-to-mesenchymal transition (NES 1.89; *p*-value < 0.0001; FDR < 0.001) were identified. Figure 3, panels A–C, shows the enrichment plots of the most affected cancer-related pathways.

## 4. Discussion

The m6A modification can alter RNA folding, stability, and molecular interactions and its dysregulation can have severe consequences on cellular function, including carcinogenesis [24]. However, methodologies to comprehensively quantify this modification have not been developed until very recently [25]. With the development of meRIP-seq/m6A-seq and then miCLIP-seq techniques, transcriptome-wide analysis of RNA modification became possible for the first time [6,7,8]. This led to the generation of high-throughput datasets for multiple cellular conditions and pathologies, including cancer [26,27]. However, these methodologies identify a significant number of false-positive m6A sites due to cross-reactivity of m6A antibodies, which can lead to misinterpretation of research results [28]. Therefore, the large datasets of potential m6A gene targets require more in-depth external validation [29].

Significant differences in global m6A modification patterns and associated RNA expression profiles between ccRCC and normal renal tissue were previously identified [23]. The aim of this work was to perform an external validation of this m6A-seq data. Expression analyses showed that 25.71% of the potential m6A-driven mRNA candidates were significantly and consistently dysregulated in ccRCC subjects. Further in-depth analyses showed that consistent dysregulation of several genes in ccRCC was present in only 11.67% of the study cohort. The low concurrence between the meRIP-seq data and mRNA expression profiles may in part be due to selection bias caused by the small number of samples (*n* = 3) in the original study.

Many studies have shown the importance of the m6A modification in RCC, although its exact role is still unclear [30]. Most investigations have focused on the components of the modification complex but downstream pathways are often not considered. Thus, unlike other cancers, there are no experimental drug approaches to date for potential m6A targets in ccRCC [31]. Small molecule METTL3 inhibitors have already shown promising results in other diseases such as acute kidney injury or myeloid leukaemia. Thus, an m6A-targeted strategy could also have a therapeutic benefit in ccRCC [32,33]. Our results show that expression of the m6A-driven key targets NDUFA4L2, NXPH4, and UMOD was significantly dysregulated in a ccRCC cohort and there was higher expression of m6A readers in ccRCC subjects, ensuring m6A target effects.

NDUFA4L2 is regulated by hypoxia inducible factor 1α (HIF1α) and transcription factor ETS domain-containing protein ELK1 and has been shown to be essential for metabolism in ccRCC [34,35]. Regulatory access via the m6A modification has not yet been elucidated but could represent promising approach for development of novel therapeutics. NXPH4, on the other hand, has not been associated with RCC carcinogenesis but may be a biomarker for bladder cancer [36,37]. In urothelial carcinoma, NXPH4 affects glycolysis and cell proliferation by maintaining the stability of NDUFA4L2 [38]. The same molecular mechanism may be occurring in ccRRC since our functional analysis suggests that hypoxia and glycolysis pathways are activated in the NNU panel. Thus, one m6A-focused therapeutic approach could be to simultaneously target NXPH4 and NDUFA4L2.

Here, we show that UMOD is downregulated in ccRCC, a finding that has been previously reported [39,40]. The product of UMOD, uromodulin or Tamm–Horsfall protein, is known to be a renal-specific protein and is currently used in clinical practice as an indicator of functional renal mass [41]. There is evidence that UMOD mutations play a regulatory role in the epithelial-to-mesenchymal transition and transformation of renal tubular epithelial cells [42]. Results from pathway analysis shows that the NNU panel is associated with the activated epithelial-to-mesenchymal transition in ccRCC tissue, which could contribute to a mechanistic model of fibrotic remodelling of the affected renal tissue during carcinogenesis. The regulatory mechanism of UMOD gene expression appears to be very complex, with involvement of HNF1B through its binding sites [43]. Until now, epitranscriptomic mechanisms for silencing UMOD gene expression have not been considered but could be of great interest for ccRCC as well as other urological (e.g., urinary tract infections, urolithiasis) and nephrological (e.g., autosomal dominant tubulointerstitial kidney disease, chronic kidney disease) diseases [41,44]. Uromodulin is a clinically accessible biomarker that is routinely measured in urine and serum. However, prospective, large-scale comparative studies in ccRCC patients would be needed to adjust reduced uromodulin levels by a correction factor that would account for impaired renal function due to other reasons [45]. It could also be conceivable that uromodulin, besides its potential as an oncogenic biomarker, could be useful in perioperative diagnostics to estimate fibrotic and atrophic renal damage due to tumour growth with associated early-stage chronic kidney disease [46,47]. Present risk stratification should be expanded, and it should be investigated whether patients with low perioperative uromodulin levels need early nephrological follow-up [48].

Regarding this study’s limitations, we need to mention that our validation is exclusively an in silico expression data analysis. In an era where enormous datasets are collected in a high-throughput manner, in-depth, detailed computational strategies for the correct identification of biomarkers are essential to avoid costly experimental studies. However, further validation of the NNU panel in the laboratory, especially for single-nucleotide resolution of their m6A marks by new orthogonal m6A detection methods such as DART-Seq or GLORI, is needed.

In summary, the NNU panel for ccRCC is a valuable source for identifying novel m6A-driven candidate genes for diagnosis and therapy. More effort is required to further validate and characterise these biomarkers by orthogonal m6A detection methods [49].

## Figures and Tables

**Table 1:** TCGA expression validation analysis data. Detailed expression information for the 35 m6A-driven defined target genes identified by Chen et al. (21). m6A-seq fold changes (fc), *p*-values, and RNA-seq fc and *p*-values were obtained from the original publication. * The “Consensus up/down” column indicates an mRNA dysregulation pattern that is consistent across the Chen and TCGA-KIRC cohorts and meets the significance criteria (*p*-value < 0.05 and fc > 2 or <0.5).

Gene Name	Chen Pattern	M6A SEQ FC	M6A SEQ *p*-Value	RNA SEQ FC	RNA SEQ *p*-Value	TCGA Pattern	TCGA FC	TCGA *p*-Value	Up/Down Consensus
FTH1	Hyper-up	522.24	<0.0001	6.62	0.0114	up	1.74	<0.0001	yes
BASP1	Hyper-up	122.49	<0.0001	59.26	<0.0001	up	1.91	<0.0001	yes
SAA1	Hyper-up	113.99	<0.0001	107.56	0.0064	up	21.51	<0.0001	yes *
IRS2	Hyper-up	94.74	<0.0001	16.48	0.0222	up	1.68	<0.0001	yes
NDUFA4L2	Hyper-up	79.53	<0.0001	82.32	<0.0001	up	55.55	<0.0001	yes *
PLOD2	Hyper-up	14.54	<0.0001	47.74	0.0351	up	3.45	<0.0001	yes *
NXPH4	Hyper-up	2.74	0.0011	53.12	0.0239	up	23.17	<0.0001	yes *
SCAF11	Hyper-up	41.00	0.0007	29.31	0.0365	up	1.01	0.7654	yes
NEPRO	Hyper-up	3.75	0.0262	9.25	0.0486	down	0.96	0.2387	no
NPAS3	Hyper-up	17.60	0.0308	11.03	0.0126	down	0.62	<0.0001	no
BMPR1A	Hyper-down	189.51	<0.0001	0.04	0.0180	down	0.73	<0.0001	yes
ZNF710	Hyper-down	114.10	<0.0001	0.15	0.0043	down	0.85	0.0017	yes
CHDH	Hyper-down	3.96	0.0039	0.09	0.0090	down	0.40	<0.0001	yes *
NDUFS7	Hyper-down	3.32	0.0169	0.06	0.0118	down	0.92	0.2739	yes
KLF11	Hypo-down	0.25	0.0028	0.09	0.0138	down	0.86	0.0053	yes
ESPN	Hypo-down	0.15	0.0022	0.01	0.0138	down	0.57	<0.0001	yes
SETBP1	Hypo-down	0.11	<0.0001	0.01	0.0022	down	0.51	<0.0001	yes
AKAP6	Hypo-down	0.07	0.0444	0.02	0.0382	down	0.72	0.0001	yes
CNTFR	Hypo-down	0.07	0.0444	0.02	0.0401	down	0.40	<0.0001	yes *
UMOD	Hypo-down	0.00	<0.0001	0.00	<0.0001	down	0.00	<0.0001	yes *
ANK3	Hypo-down	0.00	<0.0001	0.02	0.0327	down	0.39	<0.0001	yes *
NAT8	Hypo-down	0.01	<0.0001	0.04	0.0266	down	0.79	0.1651	yes
NRG1	Hypo-down	0.06	<0.0001	0.01	0.0043	down	0.97	0.7982	yes
NPR3	Hypo-down	0.10	<0.0001	0.01	0.0036	down	0.96	0.8058	yes
RERG	Hypo-down	0.22	0.0126	0.00	0.0003	up	1.07	0.4987	no
ERCC1	Hypo-up	0.11	<0.0001	22.76	0.0103	up	1.39	<0.0001	yes
FCHSD1	Hypo-up	0.07	0.0444	31.01	0.0059	up	2.46	<0.0001	yes *
FUT8	Hypo-up	0.47	0.0008	32.44	0.0376	up	1.03	0.6715	yes
G3BP2	Hypo-up	0.37	0.0398	62.38	0.0203	down	0.69	<0.0001	no
MMACHC	Hypo-up	0.17	<0.0001	33.78	0.0299	down	0.56	<0.0001	no
ALDH1A3	Hypo-up	0.16	0.0194	35.26	0.0293	down	0.37	<0.0001	no
LCORL	Hypo-up	0.13	<0.0001	41.21	0.0052	down	0.79	<0.0001	no
CHKA	Hypo-up	0.06	<0.0001	12.86	0.0079	up	1.02	0.7452	yes
REV1	Hypo-up	0.06	0.0444	291.62	<0.0001	down	1.00	0.9467	no
SRFBP1	Hypo-up	0.03	0.0037	126.92	<0.0001	down	0.86	0.0017	no

**Table 2:** Characteristics of 13 significantly upregulated gene sets based on the NNU panel. Significance criteria comprise a *p*-value < 0.05 and a false discovery rate (FDR) < 0.25. ES = enrichment score, NES = normalised enrichment score, NOM P-VAL = normalised *p*-value, and FDR Q-VAL = false discovery rate q-value. * indicates significant *p*-values.

Gene Set	Size	ES	NES	NOM P-VAL	FDR Q-VAL
HALLMARK_HYPOXIA	200	0.47	2.26	<0.0001 *	<0.0001
HALLMARK_GLYCOLYSIS	196	0.47	2.25	<0.0001 *	<0.0001
HALLMARK_EPITHELIAL_MESENCHYMAL_TRANSITION	200	0.39	1.89	<0.0001 *	0.0009
HALLMARK_COAGULATION	138	0.41	1.86	<0.0001 *	0.0014
HALLMARK_P53_PATHWAY	196	0.35	1.70	<0.0001 *	0.0030
HALLMARK_REACTIVE_OXYGEN_SPECIES_PATHWAY	49	0.45	1.70	0.0026 *	0.0029
HALLMARK_INTERFERON_ALPHA_RESPONSE	95	0.37	1.64	0.0030 *	0.0046
HALLMARK_DNA_REPAIR	148	0.36	1.64	<0.0001 *	0.0040
HALLMARK_MYOGENESIS	199	0.32	1.54	<0.0001 *	0.0106
HALLMARK_ANGIOGENESIS	36	0.40	1.42	0.0420 *	0.0298
HALLMARK_UV_RESPONSE_UP	156	0.29	1.38	0.0067 *	0.0384
HALLMARK_INTERFERON_GAMMA_RESPONSE	197	0.28	1.33	0.0070 *	0.0509
HALLMARK_XENOBIOTIC_METABOLISM	198	0.26	1.24	0.0269 *	0.0940

## Data Availability

All relevant data are included in the article and Supplementary Information Filesor can be requested from the corresponding author upon justified request.

## Supplementary Materials

The following supporting information can be downloaded at: https://www.mdpi.com/article/10.3390/diagnostics13050823/s1, Figure S1: In-depth expression stratification of the validated m6A-driven target genes of the hyper-up and hypo-down clusters.
