# Supplementary material for: An m6A-Driven Prognostic Marker Panel for Renal Cell Carcinoma Based on the First Transcriptome-Wide m6A-seq
Source: Diagnostics (Basel). 2023 Feb 21;13(5):823. doi: 10.3390/diagnostics13050823 (PMC10001021; doi:10.3390/diagnostics13050823)
Supplement: Supplementary file 1 [file diagnostics-13-00823-s001.zip › diagnostics-2223341-supplementary.pdf]

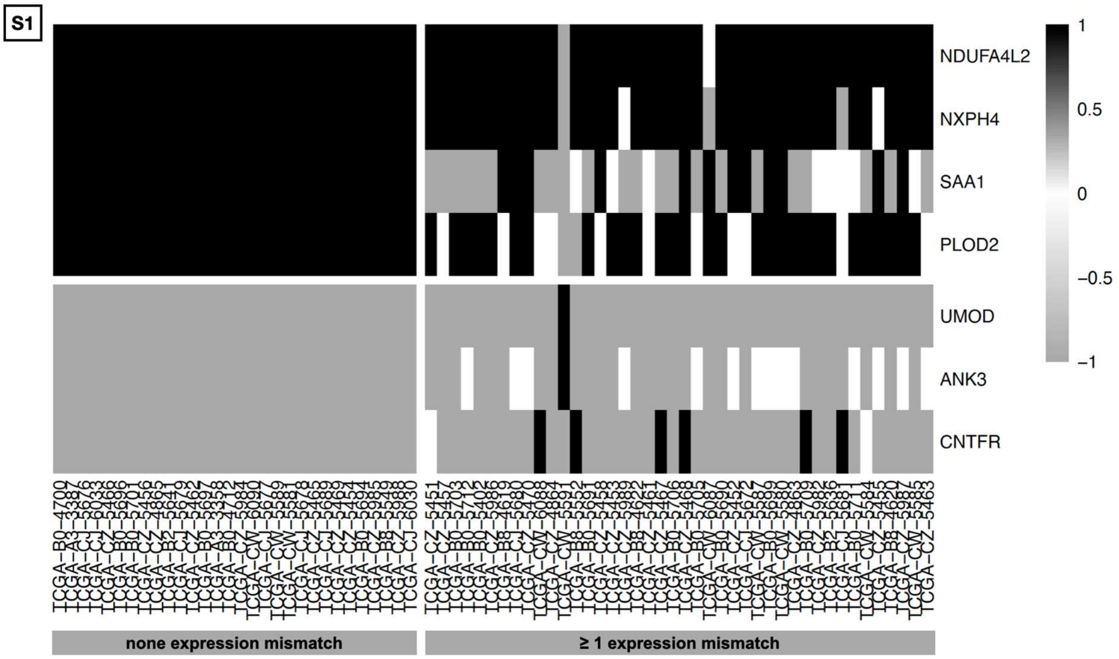

Figure S1. In-depth expression stratification of the validated m6A-driven target genes of the hyper-up and hypo-down clusters. Visualisation of binarised expression differences were based on 72 corresponding samples within the KIRC cohort. Samples with a fold change for the respective genes >1.5 were assigned as 1 (black) and samples with a fold change <0.66 were assigned as -1 (grey). Corresponding samples with neutral expression differences (fold change 0.66-1.5) are shown in white. All samples were homogenised into two groups based on the binarised results. Group 1 shows no expression mismatch according to the Chen et al. report (21) in any of the 7 identified m6A-driven target genes (left side). Group 2 comprises all corresponding samples in which at least one expression mismatch occurred in the TCGA expression stratification (right side). The visualisation shows a homogeneous and consistent expression pattern (expression deviation <10%) only for NDUFA4L2, NXPH4, and UMOD.
